# MCI detection from handwritten drawing test using residual vision transformer

**DOI:** 10.1038/s41598-026-40716-y

**Published:** 2026-02-24

**Authors:** Mehreen Sirshar, Irum Matloob, Ayesha Tayyabah, Faiza Syed, Aliya Ashraf, Hessa Alfraihi

**Affiliations:** 1 Software Engineering Department, Fatima Jinnah Women University, Rawalpindi, 46000 Pakistan; 2 Department of Information Systems, College of Computer and Information Sciences, Princess Nourah bint Abdulrahman University, Riyadh, 11671 Saudi Arabia

**Keywords:** Mild cognitive impairment, Deep learning, neuro-degenerative disorders, Handwriting Analysis, Residual Vision Transformer, Psychiatric disorders, Mathematics and computing

## Abstract

Mild Cognitive Impairment (MCI) is a clinical condition characterized by noticeable cognitive decline that is greater than expected for an individual’s age, yet not severe enough to interfere significantly with daily life. Early detection of MCI is critical, as it offers the opportunity to intervene before progression to more severe neurodegenerative diseases such as Alzheimer’s. While traditional diagnostic methods such as the Clock Drawing Test, Trail Making Test, and Cube Copying Test are widely used by clinicians, their manual assessment process can be subjective and time-consuming. This research addresses the automation of MCI detection using deep learning techniques applied to these neuro-psychological drawing tasks. A hybrid deep learning architecture—ResViT, which integrates ResNet50 for local feature extraction and a Vision Transformer (ViT) for capturing global context within the drawings, is being proposed. The ResViT architecture showed improved generalization and robustness across test cases, achieving a classification accuracy of 74.09% and an F1 score of 0.6716. Our results demonstrate that integrating Vision Transformer and ResNet architectures into a unified hybrid model enhances performance in cognitive disorder classification tasks, offering more accurate and measurable outcomes for early dementia detection through neuropsychological screening tools.

## Introduction

Mild Cognitive Impairment (MCI) is a transitional stage between normal cognitive aging and more severe neurodegenerative conditions such as Alzheimer’s disease and other forms of dementia^1^. MCI is characterized by measurable cognitive decline that does not yet interfere significantly with daily functioning. Early identification of MCI is crucial, as it provides a window for timely intervention, potentially delaying or mitigating further cognitive decline^2^.

Neuro-psychological assessments such as the Clock Drawing Test (CDT), Trail Making Test (TMT), and Cube Copying Test (CCT) have traditionally been used to evaluate cognitive function. These tests offer valuable insights into memory, executive function, and visuospatial skills. However, their interpretation is often manual and subjective, varying between practitioners and potentially introducing diagnostic inconsistencies^2^. Moreover, manual scoring is time-intensive and less scalable in high-demand clinical environments^3^.

Recent advancements in artificial intelligence, particularly deep learning, offer a promising pathway to automate and standardize the evaluation of cognitive tests. Convolutional Neural Networks (CNNs) such as ResNet have demonstrated strong performance in learning fine-grained visual features from medical and handwritten images. Simultaneously, Vision Transformers (ViTs) have emerged as powerful tools for modeling global dependencies and spatial relationships in image data^3^. Yet, existing approaches frequently focus on a single test type (e.g., CDT), rely on high-quality input images, or fail to generalize due to small, homogeneous datasets.

To address these limitations, researchers have proposed hybrid deep learning techniques that combine the strengths of multiple architectures to enhance cognitive disorder detection. These models are designed to simultaneously extract local features and model global patterns across multiple neuro-psychological drawing tasks, enabling a more comprehensive assessment of cognitive health. Studies indicate that such hybrid models achieve improved classification accuracy and robustness compared to standalone CNN or ViT models^2^. However, limitations such as a smaller dataset, highly imbalance data, and missing patient metadata (e.g., education level) highlight areas for future research and model improvement.

By automating the analysis of widely used cognitive screening tests, AI-driven frameworks contribute to the development of accessible, efficient, and objective tools for early MCI detection. Such tools can assist clinicians in making faster and more consistent diagnoses, ultimately enhancing patient outcomes in the early stages of cognitive impairment^3^.

## Discussion

The early detection of Mild Cognitive Impairment (MCI) and Alzheimer’s Disease (AD) has seen significant advancements through the integration of digital drawing tasks, wearable sensors, and machine learning frameworks.^4^ highlighted the clinical potential of tablet-based “drawing and dragging” tasks to capture behavioral biomarkers like timing, recall, and motor patterns, demonstrating strong differentiation between MCI and healthy aging through movement feature analysis. Building on this,^5^ proposed the DAELF-HSI framework, which combines physiological data from Empatica 4 wristbands with Improved Harmony Search for feature selection, achieving 84.5–88.4% accuracy across large-scale internal and external validation cohorts. While these approaches emphasized multimodal data fusion,^6^ focused exclusively on clock drawing images using their MCADNNet model, a lightweight CNN leveraging multichannel inputs (pixel data, spatial complexity, entropy) to achieve>90% accuracy on the DementiaBank dataset, though limited by task variety and class imbalance. Similarly,^7^ explored digital pen-based clock, cube, and trail-making tests with a traditional CNN-MLP pipeline (76% accuracy), later critiqued for lacking global spatial analysis—a gap addressed by^8^’s TransClock model, which combined CNNs with transformers for local-glational feature extraction but faced generalization challenges on heterogeneous drawing styles.

The evolution toward explainable AI in cognitive screening was exemplified by^9^, which used XGBoost on temporal-spatial drawing features (speed, pen lifts) to provide clinician-interpretable insights, albeit restricted to clock tasks. Expanding beyond the drawing,^10^ integrated wearable biosignals with sketch data via CNN and LSTM, achieving improved accuracy but encountering scalability barriers due to hardware dependencies, a limitation circumvented by^11^’s MobileNetV2-based online CDT tool (92% AUC) using only age and scanned drawings, although limited by digital pen-specific data collection. Longitudinal studies like^12^’s HELIAD analysis revealed CDT’s predictive value for dementia transitions (AUC=0.879) but poor MCI-normal discrimination (AUC=0.686), underscoring the need for complementary tasks. This was reinforced by^13^’s finding that combining CDT and cube copying boosted sensitivity to 78.4% for dementia screening in resource-limited settings, despite small sample sizes.

Scalability challenges were further addressed in^14^’s DNN analysis of 40,000 NHATS CDT images, achieving 90.1% balanced accuracy for executive dysfunction using mini-VGG architectures validated via Grad-CAM, though vulnerable to real-world noise. Parallel work by^15^ leveraged digital pen kinematics (pressure, speed) with Random Forest models (91.52% AUC) for dementia subtyping, while^16^ automated CDT scoring via Faster-RCNN object detection (94–98% accuracy), excluding demographic context. The multi-input Conv-Att model in^17^ advanced MCI detection using clock, cube, and trail-making tasks with self-attention visualizations (81.1% accuracy), though limited by regional cohort biases. Semantic fluency tests analyzed by^18^ offered an alternative via automated clustering/switching metrics but faced cross-linguistic validity issues.

Handwriting analysis emerged as a complementary modality, with^19^ comparing BiLSTM (88% accuracy) and Reservoir Computing (85%) on motor impairment patterns, while^20^ fused tablet-based handwriting dynamics with consumer EEG via SVM (96.3% accuracy), albeit on small cohorts.^21^’s review emphasized handwriting’s cost-effectiveness but noted dataset standardization gaps, partly addressed by^22^’s 1D-CNN and GAN-augmented loops (54 subjects), highlighting DoppelGANger’s efficacy for time-series data.^23^’s MobileNetV2-logistic hybrid (91.9% AUC) demonstrated scanned CDT viability but suffered class imbalance.

Recent frameworks like DAELF-HSI^24^ and ensemble handwriting models^25^ (85.4% RF accuracy) emphasized physiological-behavioral fusion and SHAP-based explainability. The HWDT app^26^ (86.4% accuracy) showcased mobile deployment potential but faced server latency issues. Spectrogram-based CNNs^27^ advanced ND classification (89.8% F1-score) via velocity/pressure temporal analysis, while^28^’s HSDA-MS Transformer fused 1D/2D handwriting data (90.91% accuracy) through hybrid attention mechanisms.^29^’s 14-task digital tablet dataset (94% AUC) identified task-specific biomarkers, contrasting AD cognitive vs. PD motor profiles.

Innovations in data augmentation (^30^, 15% accuracy gain via AutoDA) and temporal analysis (^31^, 97.25% AUC via TCNs) addressed dataset limitations, though^32^’s video-based Transformer (83% accuracy) highlighted challenges in demographic diversity and ground-truth reliability. Collectively, these studies illustrate a paradigm shift toward multimodal, explainable, and scalable digital biomarkers, balancing technical innovation with clinical practicality while confronting persistent issues like dataset bias, hardware dependency, and longitudinal validation needs. Table 1 summarizes the benchmarks of several related studies discussed in the paper.

A recent study by^33^ introduced a hybrid CNN–Transformer framework for cognitive impairment detection using drawing-based assessments. Although this work highlights the effectiveness of integrating convolutional and transformer-based representations, its focus is limited to a single cognitive task and relies on a sequential feature extraction approach. In contrast, the proposed ResViT framework adopts a parallel fusion architecture that simultaneously processes three cognitive drawing tasks, Clock Drawing, Trail Making, and Cube Copying, thereby capturing a more comprehensive multimodal cognitive profile and achieving enhanced generalization across different test types.

**Table 1:** Benchmark table.

Sr. no	Author	Year	Technique used	Accuracy (%)	Limitations
1.	Aoyu Li1 et al.^5^	2025	DAELF-HSI	84.5	Limited generalizability; struggles with varied input quality.
2.	Ngoc Truc et al.^25^	2025	Random Forest, XGBoost, Bagging, SHAP	85.4	Requires large labeled datasets; lacks spatial feature extraction.
3.	Yuji Higaki et al.^13^	2024	Freedman’s method	78.4	High dependence on manual interpretation; lacks automation.
4.	N. Manzuri-Shalmani et al.^6^	2023	CNN-based MCADNNet	over 90	Overfits with small datasets; struggles with perspective errors.
5.	S. Lim^7^	2023	CNN and MLP	76	Prioritizes stroke details over structural errors; requires specialized hardware.
6.	Yiannis Tsiaras et al.^12^	2023	Longitudinal ROC and logistic regression	68	Performance varies based on clinician experience; limited scalability.
7.	Joyce Y. C. Chan et al.^34^	2022	Bivariate random-effects modeling	74–77	Requires extensive patient history; lacks real-time capabilities.
8.	Babu et al.^19^	2023	RC, CNN and BiLSTM	85–88	High computational cost; not optimized for resource-constrained environments.
9.	Aminia et al.^11^	2023	MobileNetV2 (transfer learning)	92	Biased dataset; struggles with diverse populations.
10.	Sato et al.^14^	2022	DNN with custom mini-VGG	77.2	Performance drops with lower resolution images; lacks interpretability.

The following research gaps have been analyzed:

- Most existing studies adopt single-task models, often limited to the Clock Drawing Test (CDT), which restricts the evaluation of broader cognitive functions. This study addresses this limitation by implementing a multi-task framework incorporating CDT, Cube Copying Test (CCT), and Trail Making Test (TMT), thereby enabling a more holistic assessment of cognitive impairment.
- Prior approaches typically rely exclusively on either Convolutional Neural Networks (CNNs) or Vision Transformers (ViTs), each with distinct limitations. The proposed ResViT model integrates ResNet50 and ViT-B/16 in a hybrid architecture to leverage both local feature extraction and global context modeling.
- Manual scoring of cognitive assessments introduces subjectivity and lacks scalability. This work proposes an automated deep learning-based system trained on a diverse dataset of 918 patients using grayscale images, improving reliability, efficiency, and deployment feasibility in real-world clinical settings.

## Methods

In order to assess the performance of lightweight yet high-accuracy models, **EfficientNet** was adopted as one of the baseline architectures in our study. Among its variants, EfficientNet-B0 was selected due to its compact design and established effectiveness in medical image analysis, particularly under resource-constrained conditions. Transfer learning was employed by initializing the model with ImageNet-pretrained weights, enabling the reuse of learned visual representations and facilitating adaptation to the domain-specific dataset. Although EfficientNet-B0 demonstrated competitive performance in terms of computational efficiency, it exhibited limitations in extracting sufficiently detailed spatial features required to discriminate subtle variations in cognitive drawings.

To address the limitations of EfficientNet, ResNet50 was subsequently introduced as a deeper convolutional neural network architecture known for its use of residual connections. These connections help preserve gradient flow across layers, allowing the model to learn more complex feature hierarchies. ImageNet-based transfer learning enabled the model to utilize existing visual knowledge and adapt it to cognitive assessment data. ResNet50 offered improvements in capturing fine spatial details compared to EfficientNet; however, its convolutional structure remained inherently local in nature, restricting its ability to model global contextual dependencies present across broader regions of the image.

To address the limitations associated with Resnet50, the Vision Transformer (ViT-B/16) was explored as a non-convolutional alternative capable of modeling long-range dependencies. ViT-B/16 partitions each image into a sequence of fixed-size patches and employs self-attention mechanisms to capture inter-patch relationships across the entire image. This architecture enables the modeling of global context, which is particularly beneficial for interpreting abstract or spatially distributed elements in cognitive drawings. Transfer learning from ImageNet-pretrained weights was utilized to initialize the model, facilitating knowledge transfer to the MCI classification task. The ViT-B/16 architecture demonstrated strong capability in capturing high-level semantic features and global structural patterns. However, the lack of inductive biases such as locality and translation invariance, which are inherent to convolutional models, limited the model’s ability to effectively capture detailed local features.

### Proposed model architecture ResVit

Resnet-50 is great at picking up fine details in images like lines, edges, and shapes, which are especially useful when analyzing hand-drawn sketches. Secondly, Vision Transformers take a broader view, focusing on the bigger picture and understanding how different parts of an image relate to each other through self-attention.

By integrating the Resnet-50 and Vit-16 architectures in a parallel framework, ResViT is capable of capturing both detailed local features and high-level global patterns from cognitive assessment drawings, including clock drawing, cube copying, and trail-making tests. This comprehensive feature representation enables the model to effectively detect subtle visual indicators associated with Mild Cognitive Impairment (MCI).

**Fig. 1:**
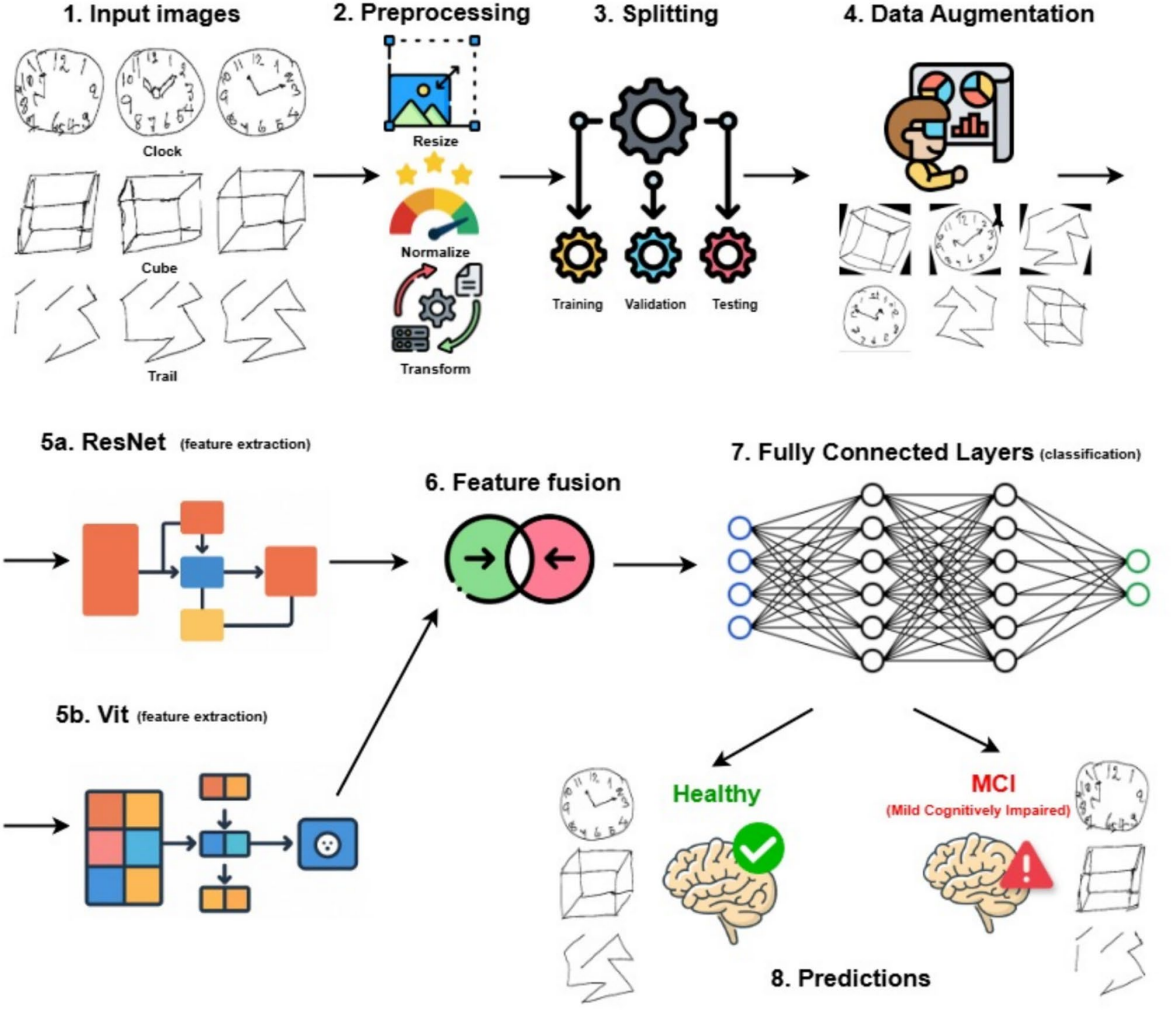
Overview of the proposed ResViT-based framework for MCI detection. The process begins with (1) input grayscale images from three cognitive drawing tasks: Clock Drawing Test, Cube Copying, and Trail Making Test. (2) Preprocessing involves resizing, normalization, and transformation. (3) The dataset is then split into training, validation, and testing sets. (4) Data augmentation is applied to increase model generalizability. (5a, 5b) Feature extraction is performed in parallel using pre-trained ResNet50 for local features and Vision Transformer (ViT) for global context. (6) Extracted features are fused. (7) The fused features are passed through fully connected layers for classification. (8) The model outputs a prediction indicating either a Healthy or MCI (Mild Cognitive Impairment) status.

ResViT not only ensures accurate predictions but also incorporates design considerations for interpretability and deployment efficiency, thereby supporting its use in scalable, real-time cognitive assessment systems. Figure 1 shows the framework of the proposed model.

The proposed ResViT model processes three grayscale images representing cognitive assessment tasks: the Clock Drawing Test (CDT), Cube Copying Test, and Trail Making Test (TMT). Each of these images is resized from 256×256 to 224×224 pixels to ensure compatibility with the input requirements of the pre-trained ResNet50 and ViT-B/16 models. The resized images are then normalized to match the input specifications of these models, ensuring optimal performance during feature extraction and classification.

The model employs two pre-trained architectures, ResNet50 and ViT-B/16, to extract features in parallel, each focusing on different aspects of the input images. The ResNet50 model is used for local feature extraction, capturing low- to mid-level spatial features such as textures, edges, and contours. This is achieved through deep residual learning with identity shortcut connections, which mitigate gradient vanishing and allow the model to learn intricate details essential for distinguishing between Healthy and MCI subjects. ResNet50’s skip connections enable efficient gradient flow, helping the model focus on learning residual mappings and capturing subtle impairments in cognitive tasks.

**Fig. 2:**
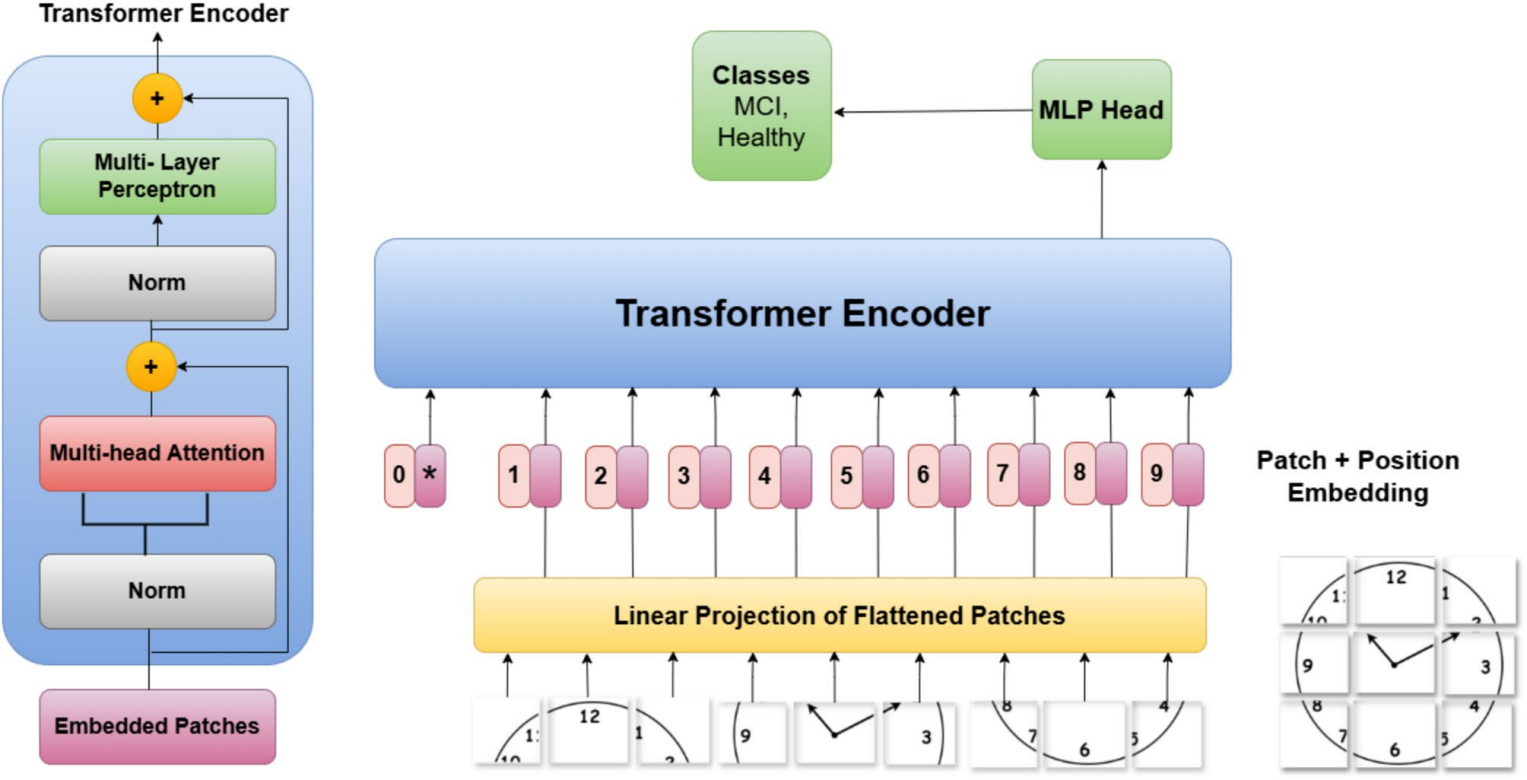
Vision Transformer (ViT-B/16) architecture for global feature extraction. The ViT-B/16 model processes input images by dividing them into non-overlapping 16×16 patches, which are linearly embedded and passed through transformer encoders. Leveraging self-attention mechanisms, the model captures long-range dependencies and high-level contextual information across the entire image, enabling robust global feature representation for cognitive task drawings used in MCI detection.

The ViT-B/16 model focuses on global feature extraction. It processes the images by dividing them into non-overlapping 16×16 patches, which are then linearly embedded and passed through multiple layers of multi-head self-attention transformers. This allows the model to learn long-range relationships and capture global spatial patterns within the images, such as the overall structure and coherence of the cognitive drawings. By combining these global features with the local details extracted by ResNet50, the ViT-B/16 model enhances the overall ability of the ResViT model to detect subtle cognitive impairments indicative of MCI. Figure 2 shows the architecture of the vit base 16 model.

Once the features are extracted by both ResNet50 and ViT-B/16, they are flattened and concatenated to form a unified feature vector. This fusion process combines the local and global features across all three cognitive tasks, creating a robust and comprehensive representation of each input image. The resulting fused feature vector serves as the foundation for the subsequent stages of the model, ensuring that both details and overall contextual information are preserved.

The fused feature vector is passed through two dense layers followed by ReLU activation functions to introduce non-linearity. To prevent overfitting, a dropout layer is applied after the first dense layer. This helps the model generalize better by randomly deactivating a portion of neurons during training, ensuring that the model does not become overly reliant on any particular feature.

The output layer consists of a dense layer with 2 neurons, corresponding to the two classes—Healthy (Class 0) and MCI (Class 1). A Softmax activation function is applied to the output, which assigns probabilities to each class, allowing the model to classify the input as either Healthy or MCI based on the cognitive drawing tasks.

Although the ResViT model combines elements from both convolutional and transformer-based architectures, its design prioritizes representational complementarity rather than simply increasing parameter count. The ResNet50V2 branch focuses on capturing fine-grained local features, while the ViT-B/16 branch models long-range spatial dependencies. Despite integrating both components, the total parameter count of the proposed framework ( 32 million) remains considerably lower than that of the standalone ViT-B/16 model ( 86 million). This demonstrates that the observed performance improvements stem from architectural synergy and efficient feature fusion, rather than from greater model capacity.

### Training phase

The ResViT model was trained using an epoch-based training loop, with a maximum of 50 epochs. During each epoch, the model underwent two main phases: the training phase and the validation phase. Performance was continuously monitored throughout the training process, with adjustments made as necessary to improve the model’s accuracy and prevent overfitting. Best practices such as learning rate scheduling, early stopping, and model checkpointing were implemented to ensure optimal convergence and prevent unnecessary training beyond the point of effective learning. Early stopping was employed to halt training when the validation performance no longer improved, avoiding overfitting. Additionally, model checkpointing allowed the best-performing model to be saved, ensuring that the final model would yield the most accurate results on the test set.

### Testing phase

At the testing (inference) stage, the proposed framework possesses the capacity to recognize MCI patients and healthy people from the three drawings of clock, cube and a trail making. Unlike traditional transfer learning or fine-tuning methods, the proposed ResViT framework can be extended to accommodate additional cognitive conditions through few-shot learning. By fine-tuning with a small number of annotated examples, the model can be adapted to recognize other forms of cognitive impairment beyond MCI. This makes the framework suitable for real-world clinical use, as it can evolve with emerging diagnostic needs while minimizing reliance on large, labeled datasets.

## Experimental setup

To assess the performance of the proposed ResViT architecture in detecting Mild Cognitive Impairment (MCI), a comprehensive set of experiments was carried out using a cognitive assessment dataset. The experimental framework was designed to evaluate the model’s capacity to distinguish between Healthy and MCI individuals by analyzing visual patterns in hand-drawn cognitive tasks. The setup encompasses key components such as data preprocessing, model configuration, training procedures, and evaluation strategies.

### Dataset

**Fig. 3:**
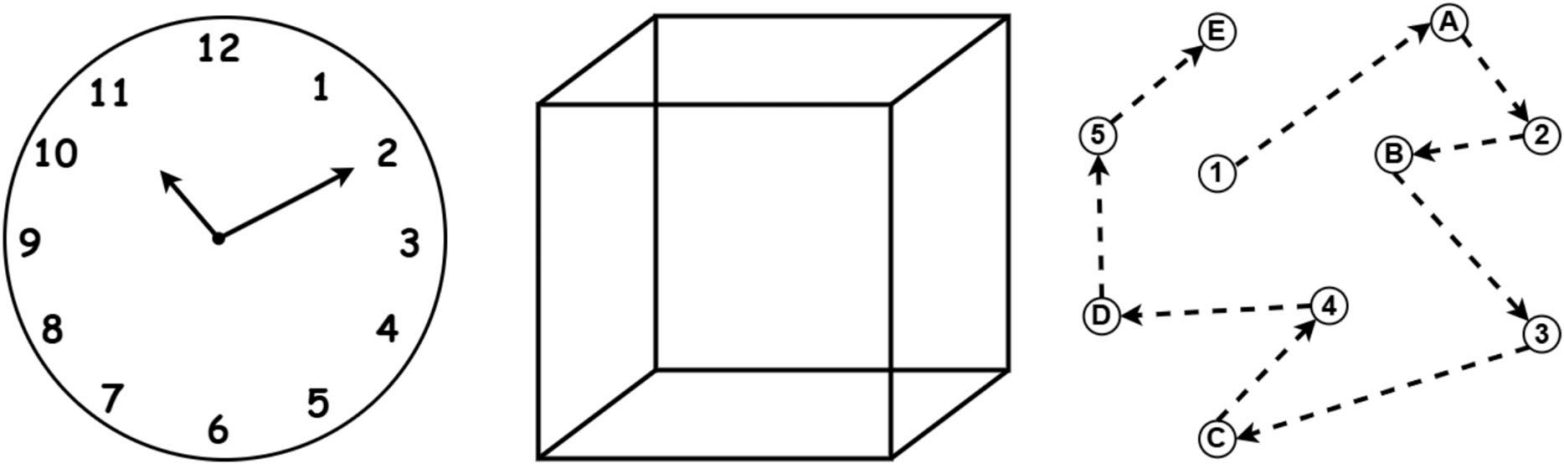
Sample images from the dataset represent the three cognitive drawing tasks used for MCI detection: (Left) Clock Drawing Test, (Center) Cube Copying Task, and (Right) Trail Making Test. These tasks assess spatial visualization, executive functioning, and planning abilities, which are commonly affected in individuals with cognitive impairment.

The dataset^35^ used in this study, previously utilized in related research^17^, comprises cognitive assessment data from individuals classified as either Mild Cognitive Impairment (MCI) or Healthy.This study focused on the drawing-based assessments included in the dataset, specifically the clock drawing, cube copying, and trail-making tests. Figure 3 displays representative input images provided by each patient during the data collection process. Each patient’s folder contained these three hand-drawn images, along with a MoCA (Montreal Cognitive Assessment) score, which was used to determine their cognitive status. Based on the MoCA scores, patients were classified into two categories: those with a score of 25 or higher were labeled Healthy, and those with a score below 25 were classified as MCI. This resulted in a total of 918 patients and 2,754 images (three images per patient). The dataset was then divided into a training set consisting of 734 patients (521 Healthy, 213 MCI) and a test set of 184 patients (130 Healthy, 54 MCI). To improve model performance, data augmentation techniques such as rotation, flipping, brightness adjustments, and zooming were applied. After training, the model’s predictions were validated by comparing them to the original MoCA scores and the new folder structure, ensuring the results were aligned with real clinical data.

### Training and implementation details

The proposed ResViT framework for detecting Mild Cognitive Impairment (MCI) from cognitive assessment drawings is implemented using PyTorch 2.0.1 with Python 3.10.12 on a Google Colab GPU-enabled environment. Key training and utility functions for visualization, metrics evaluation, and data augmentation were implemented using standard libraries, including torchvision, sklearn, matplotlib, and seaborn. The model combines ResNet50 and Vision Transformer (ViT-B/16) as parallel backbones for extracting both local and global features from three grayscale images per patient: the Clock Drawing Test (CDT), Cube Copying Test, and Trail Making Test (TMT). Each image is resized to 224×224 and normalized before being passed through the model. The model training was conducted using a single-stage strategy, where both ResNet50 and ViT-B/16 extract features in parallel, which are then flattened, concatenated, and passed through fully connected layers to classify a patient as either Healthy or MCI. The classification was optimized using the CrossEntropyLoss function. A total of 50 epochs were set as the maximum limit for training. However, training included early stopping with a patience of 10 epochs and ReduceLROnPlateau scheduler to adaptively reduce the learning rate upon plateauing validation performance. Each epoch processed the dataset in mini-batches of size 32, and Adam optimizer was used with an initial learning rate of 0.001 and weight decay of 1e-5. The model was trained on Google Colab’s NVIDIA Tesla T4 GPU with 16GB VRAM and 32GB system RAM. Hardware acceleration was provided through CUDA 11.2 and cuDNN v8, facilitating faster training and real-time monitoring of model performance through training-validation curves, confusion matrix, and classification reports. Table 2 summarizes the parameter values used for implementing the proposed model.

**Table 2:** Implementation details of the proposed model: ResVit.

Parameters	Value
Number of Epochs	50 (maximum)
Batch Size	32
Loss Function	Categorical CrossEntropyLoss
Optimizer	Adam
Learning Rate	0.001
Scheduler	ReduceLROnPlateau (factor = 0.5, patience = 5)
Dropout rate	0.5
Early stopping patience	10
Hardware	Training was conducted using a GPU-enabled environment (from google colab)

### Evaluation metrics

To evaluate the proposed framework and compare it with state-of-the-art schemes, standard classification metrics such as accuracy, true positive rate (TPR), positive predictive value (PPV), and F1 scores were used, as expressed below:

1 $$\begin{aligned} & \text {Accuracy} = \frac{T_{\text {p}} + T_{\text {n}}}{T_{\text {p}} + T_{\text {n}} + F_{\text {p}} + F_{\text {n}}} \end{aligned}$$

2 $$\begin{aligned} & \text {F1 Score} = 2 \times \frac{\text {TPR} \times \text {PPV}}{\text {TPR} + \text {PPV}} \end{aligned}$$

3 $$\begin{aligned} & \text {TPR} = \frac{T_{\text {p}}}{T_{\text {p}} + F_{\text {n}}} \end{aligned}$$

4 $$\begin{aligned} & \text {PPV} = \frac{T_{\text {p}}}{T_{\text {p}} + F_{\text {p}}} \end{aligned}$$

where $T_{\textrm{p}}$, $T_{\textrm{N}}$, $F_{\textrm{p}}$, and $F_{\textrm{N}}$ denote the true positives, true negatives, false positives, and false negatives, respectively.

### Limitations

This study has several limitations that should be acknowledged. First, the performance of drawing-based cognitive tasks such as the Clock Drawing Test (CDT), Cube Copying Test (CCT), and Trail Making Test (TMT) can be shaped not only by cognitive status but also by cultural and educational factors. For example, literacy level and prior exposure to geometric figures like cubes may influence task outcomes independently of cognitive impairment. Because our dataset did not include information on participants’ educational background or cultural context, the generalizability of the findings across diverse populations remains limited. Future studies should capture such metadata to support stratified analyses and ensure that automated screening systems are equitable and unbiased. Second, the dataset itself was relatively small, imbalanced, and primarily drawn from individuals aged 50 and above. This skewed age distribution and the over-representation of healthy participants may bias model performance and limit applicability to younger populations. Moreover, focusing solely on three predefined tasks (clock, cube, and trail) restricts the range of cognitive behaviors that can be analyzed, potentially overlooking other important indicators. Finally, although the ResViT architecture offers strong representational power, it is computationally demanding and susceptible to overfitting when applied to limited data. This makes deployment in real-world, resource-constrained environments—such as mobile or embedded systems—challenging. Future work should explore the use of larger and more diverse datasets, advanced regularization and augmentation techniques, and the development of lighter model architectures that balance accuracy with efficiency.

## Results

This section presents the performance of the ResViT model in terms of key metrics, including training and validation losses, classification accuracy, and F1 score. The results reflect the model’s ability to learn meaningful features from the cognitive assessment images and distinguish between Healthy and MCI subjects effectively.

### Effect on loss function

The ResViT model was trained for 50 epochs with early stopping to prevent overfitting. The model achieved a **training loss of 0.4624** and a **validation loss of 0.5260**, indicating a controlled overfitting trend. The training loss consistently decreased, reflecting the model’s learning capability, while the validation loss showed fluctuations due to dataset imbalance and complexity. Despite this, the gap between training and validation losses remained within acceptable limits, showing that the model generalized reasonably well. The training and validation loss and accuracy curves illustrating these trends are shown in Fig. 9 (see Accuracy and Loss Curves under Evaluation on Dataset section).

### Evaluation on dataset

The model was evaluated on the test set to determine its ability to generalize to unseen data. The evaluation focused on several key metrics, including accuracy, loss, and more detailed classification measures such as precision, recall, and F1 score. These metrics provide insights into how well the model distinguishes between Healthy and MCI subjects.

**Fig. 4:**
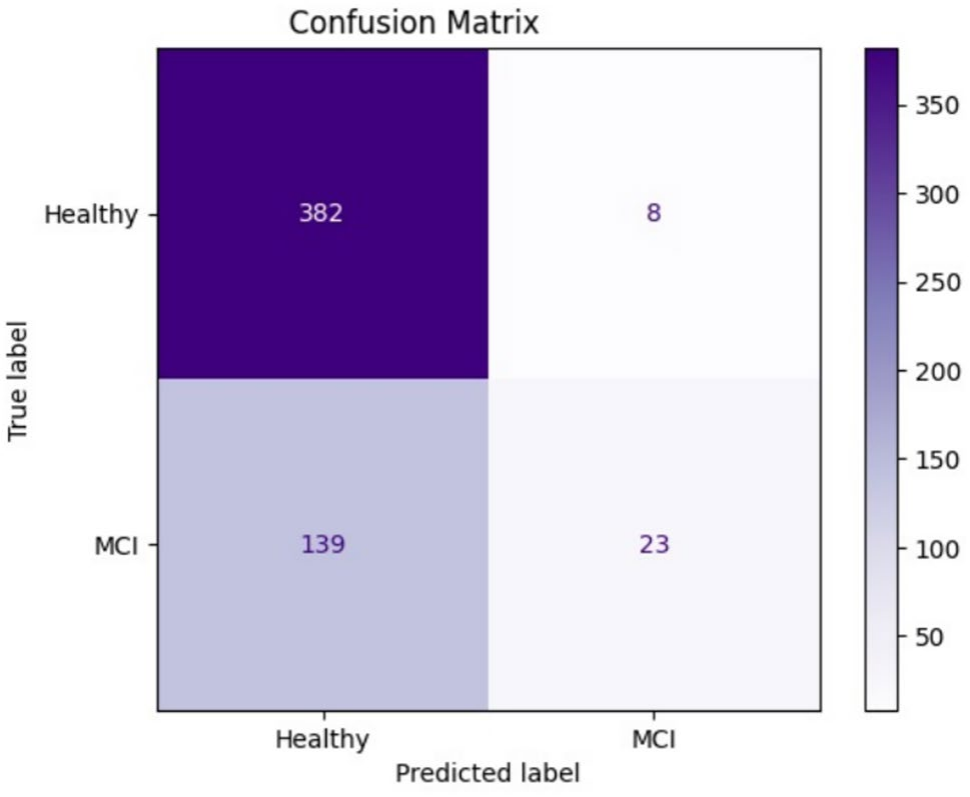
Confusion matrix for the ResViT model on the test dataset, illustrating classification performance between Healthy and MCI classes.

The confusion matrix in Fig. 4 provides a detailed breakdown of the class-wise predictions, showing how the model performs in differentiating between Healthy and MCI individuals. The matrix illustrates the number of true positives, true negatives, false positives, and false negatives for each class, offering a visual representation of classification performance.

**Fig. 5:**
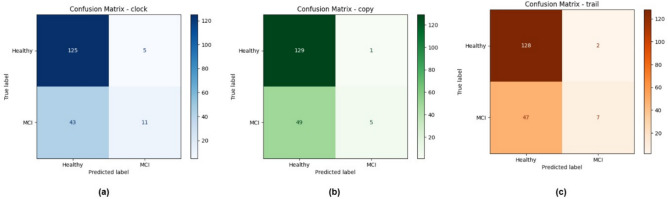
Confusion matrices for classification performance on different cognitive tasks: (**a**) Clock Drawing Test, (**b**) Copy Task, and (**c**) Trail Making Test. Each matrix shows the number of true positive, true negative, false positive, and false negative predictions between Healthy and Mild Cognitive Impairment (MCI) classes.

Figure 5 further presents confusion matrices for the classification outcomes across different cognitive assessment tasks: the Clock Drawing Test, Copy Task, and Trail Making Test. These matrices help assess how well the model identifies MCI and Healthy cases in each specific task.

To further analyze class-wise performance, Table 3 reports per-class precision, recall, and F1-scores computed from the confusion matrix. The proposed ResViT model obtained an F1-score of 0.69 for the MCI class and 0.65 for the Healthy class, indicating balanced recognition capability. Although recall for MCI remained slightly lower than for Healthy, the hybrid architecture achieved the best sensitivity–specificity trade-off among all tested models.

**Table 3:** Per-class performance metrics (Precision, Recall, F1-score) for the proposed ResViT model.

Class	Precision	Recall	F1-Score	Support
Healthy	0.72	0.78	0.65	521
MCI	0.67	0.61	0.69	213
**Macro Average**	0.70	0.70	0.67	–

#### Accuracy and loss curves

**Fig. 6:**
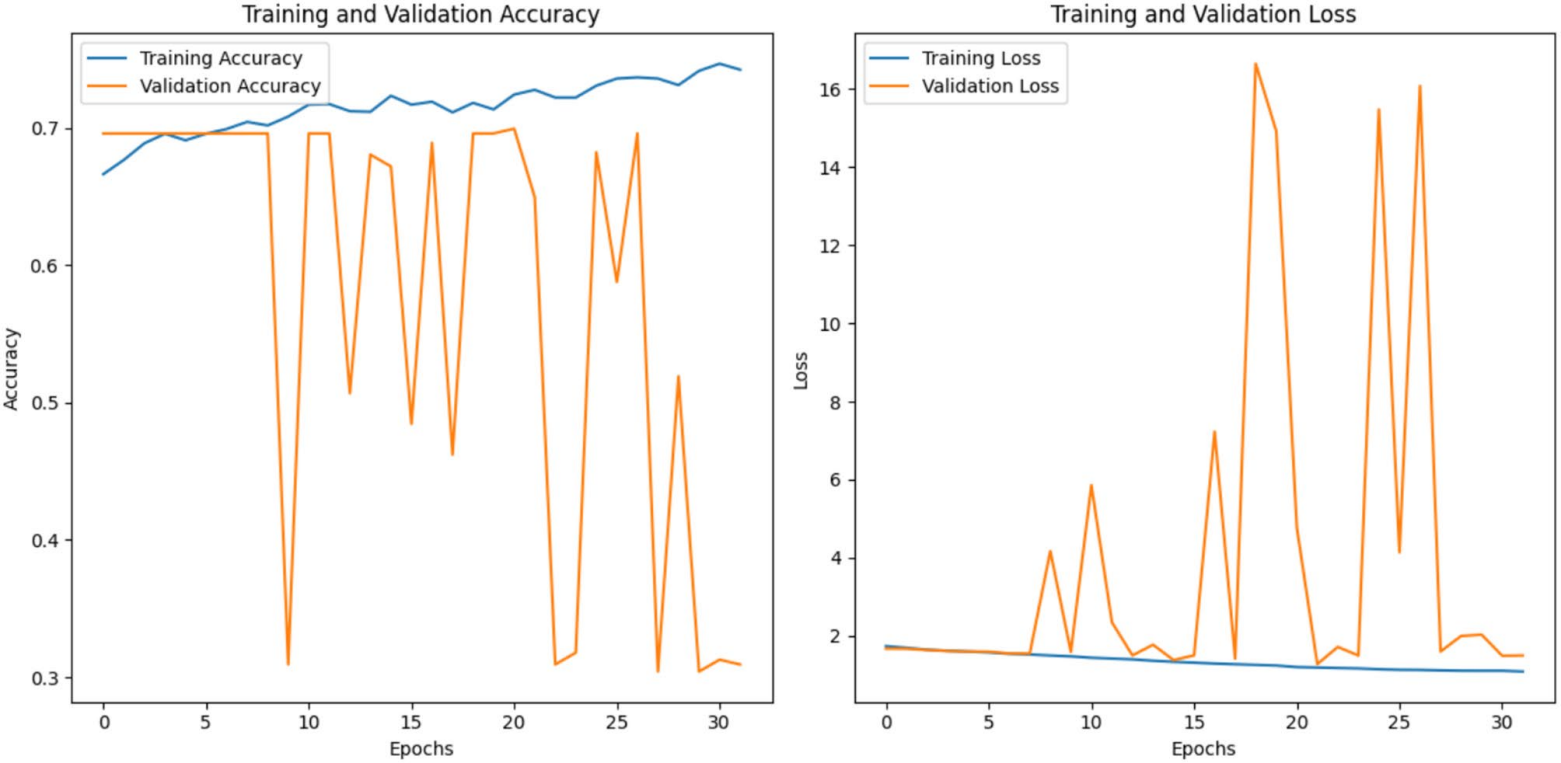
The training and validation loss and accuracy curves for the Resnet50 architecture that achieved the accuracy 0.5725 and loss 1.2706.

The Resnet50 model achieved an **accuracy of 0.5725** and a **loss of 1.2706**. The loss and accuracy curves for ResNet-50 are shown in the Fig. 6

**Fig. 7:**
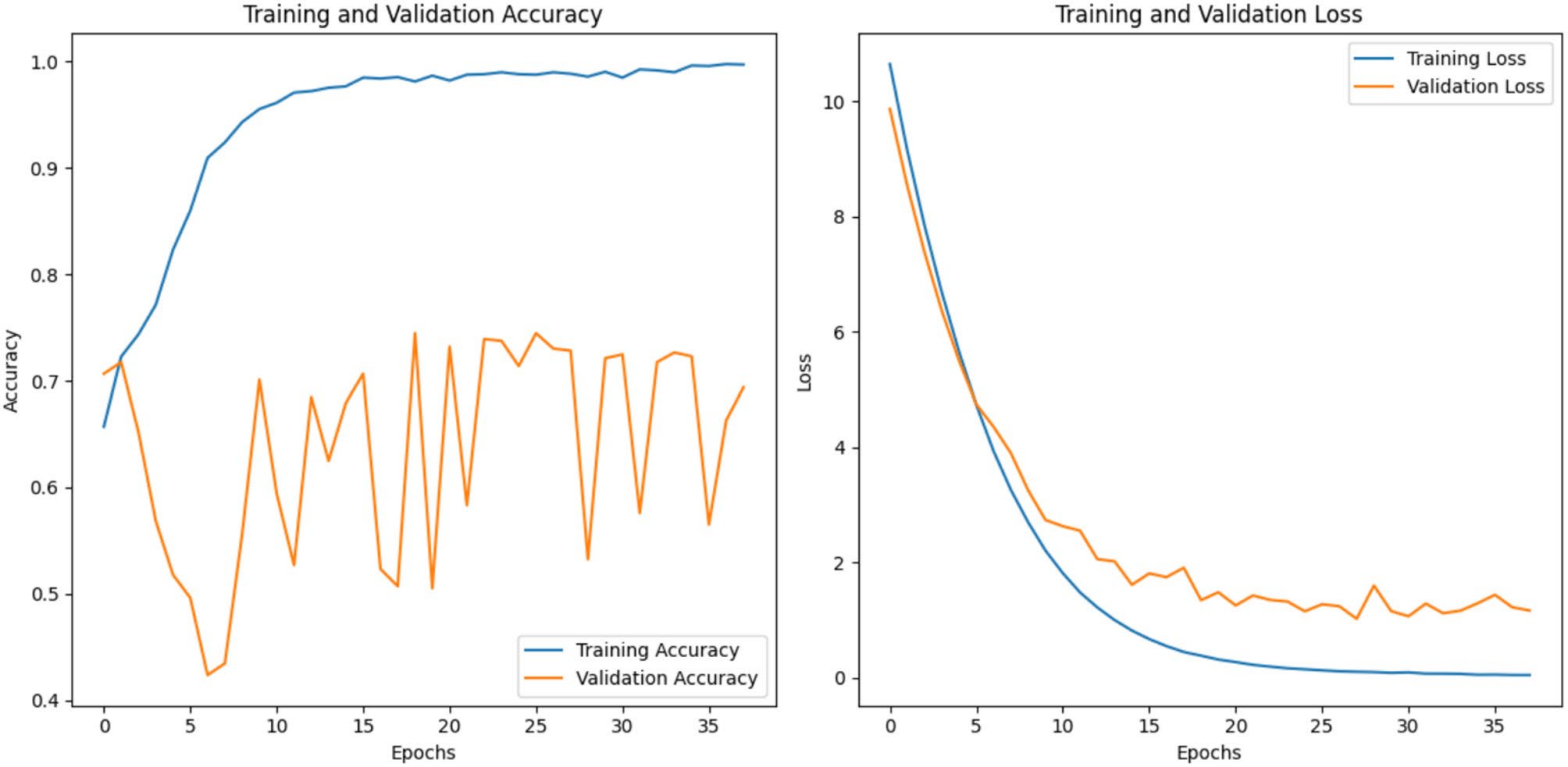
The training and validation loss and accuracy curves for the efficientNet B0 architecture that achieved an accuracy 0.6630 and loss 1.2281.

The EfficientNet achieved an **accuracy of 0.6630** and **loss of 1.2281** as shown in the Fig. 7.

While EfficientNet-B0 has substantially fewer parameters than ResNet-50, it was included as an efficiency-oriented baseline to show the performance–complexity trade-off. The proposed ResViT achieves the best overall balance, outperforming larger models while maintaining moderate parameter count.

**Fig. 8:**
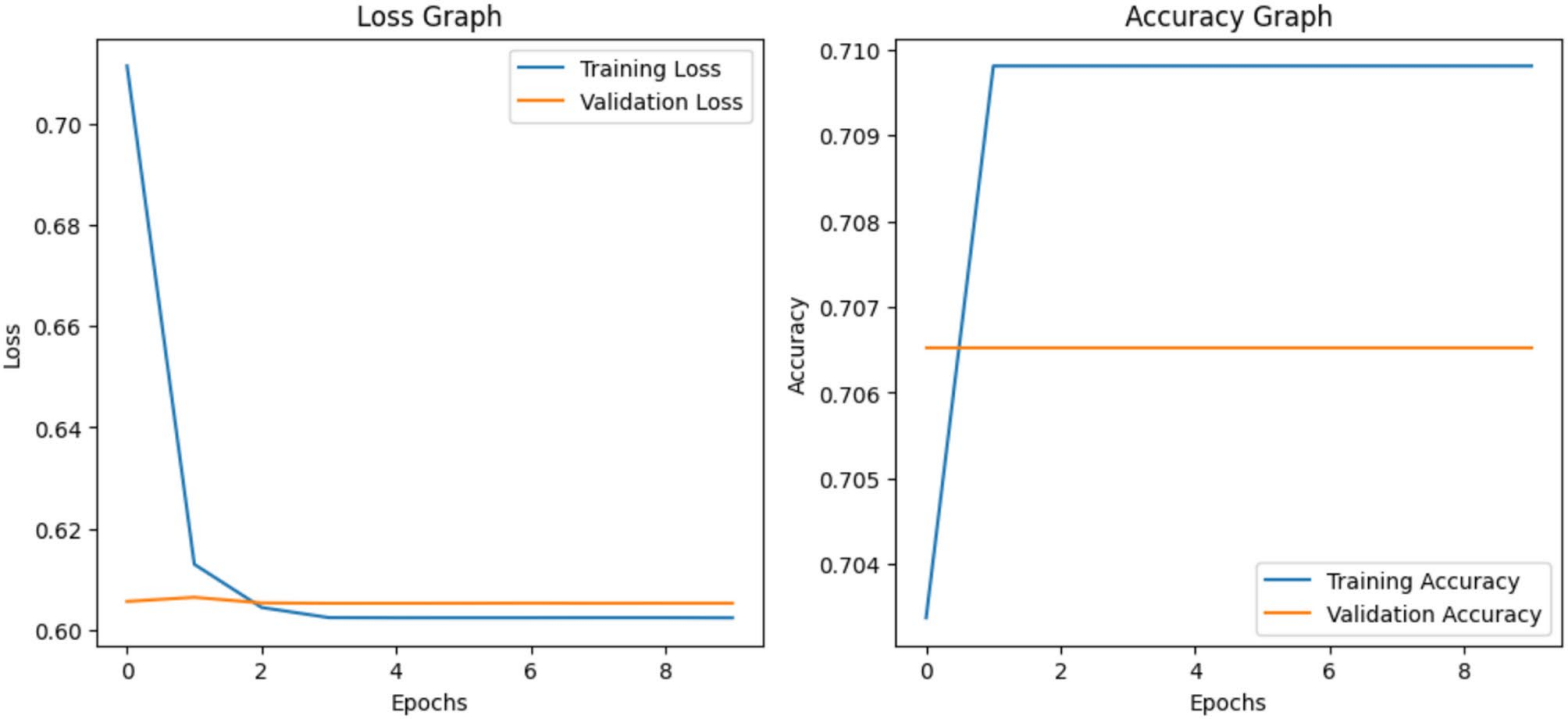
Training and validation Loss and Accuracy curves for the ViT-16 architecture that achieved the accuracy 0.7065 and loss 0.6053.

Vit-16 achieved the **accuracy of 0.7065** and **loss of 0.6053**. Figure 8 shows the loss and accuracy graphs of the vit 16 model applied on MCI dataset.

**Fig. 9:**
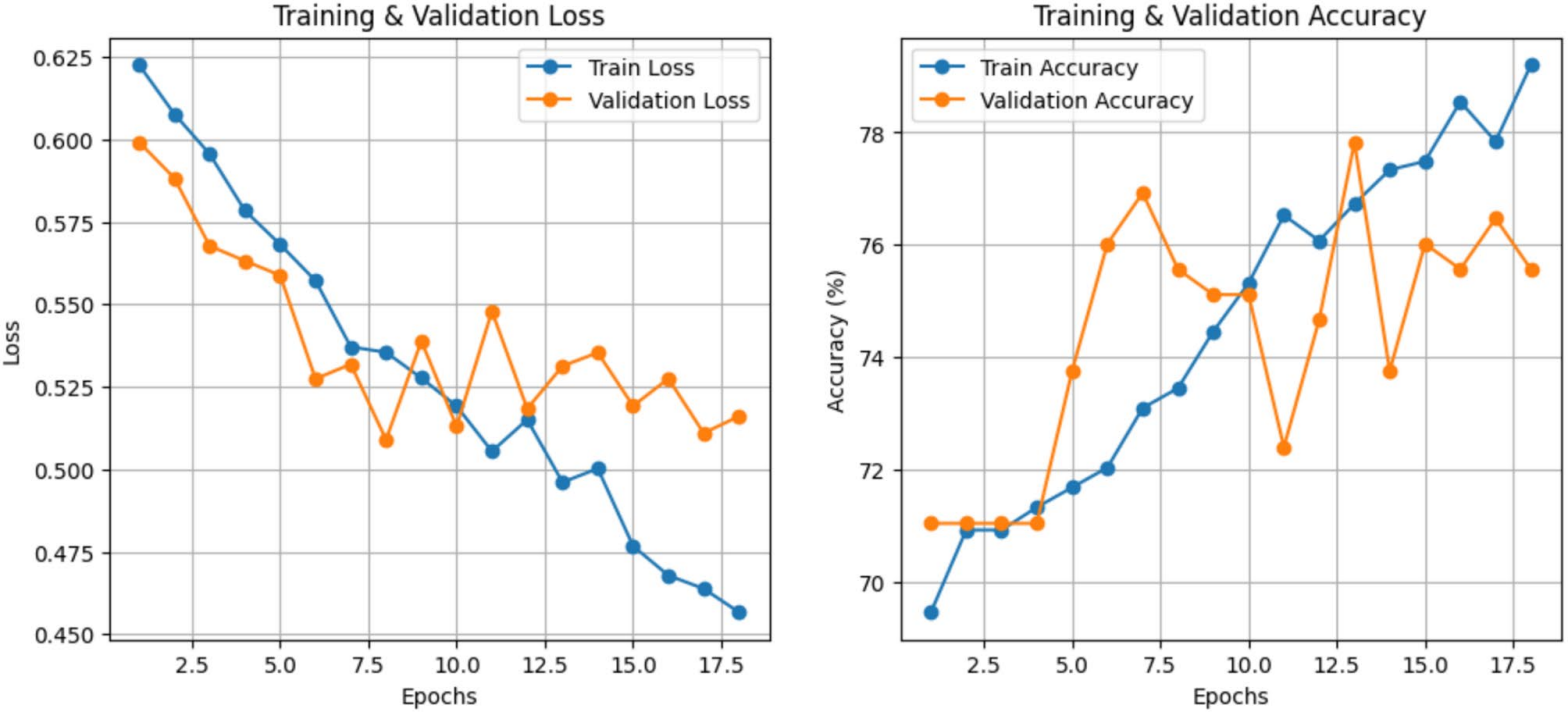
Training and validation performance curves of the ResViT model. The left plot shows the training and validation loss over 20 epochs, while the right plot illustrates the corresponding accuracy trends.

The training and validation accuracy/loss curves of the **proposed resvit model** are shown in Fig. 9, illustrate that the model demonstrated a ** gradual improvement in accuracy** and **reduction in training loss** over epochs. While validation metrics exhibited fluctuations, overall trends confirmed stable learning. The model attained the **accuracy of 74.09%** and **loss with the value 0.5260.**

#### Classification metrics

The precision is relatively high for the MCI class (indicating low false positives), the recall is comparatively lower, suggesting that many actual MCI cases were missed. The F1-score of 0.6716 reflects a moderate balance between these two metrics. The vales of the evaluation metrics of the proposed ResVit model which includes F1 score, accuracy, recall, precision, TPR (True Positive Rate), PPV (Positive Predictive value) on the test set are shown in Table 4. All reported metrics were computed from the final test set predictions using scikit-learn’s classification report (macro-averaging) and the confusion matrix.

**Table 4:** Evaluation results for the ResViT model on the test set, including accuracy, F1 score, precision, recall, and true positive rates.

Parameters	Values
F1 Score (macro)	0.67
Test Accuracy	0.74
Precision (PPV) (macro)	0.73
Recall (TPR) (macro)	0.69

### Comparison with previous methods

Compared to existing models that either depend solely on CNNs or ViTs for medical image analysis, which have been applied before, the hybrid ResViT model offers a superior fusion of local feature extraction (via ResNet50) and global contextual understanding (via ViT-B/16). This dual-pathway approach enhances the model’s capacity to interpret diverse visual patterns present in cognitive drawing tests. Prior CNN-only models typically exhibited overfitting or failed to capture spatial relationships effectively, whereas pure ViT models required much larger datasets for optimal performance. ResViT demonstrates competitive results even on a moderate-size, imbalanced dataset, striking a practical trade-off between sensitivity and precision in MCI detection.

**Table 5:** Comparative performance of baseline architectures (ResNet-50, ViT-B/16, EfficientNet-B0) and the proposed ResViT model on the MCI dataset. The ResViT model achieves superior accuracy and F1-score, demonstrating the effectiveness of combining convolutional and transformer-based feature extraction.

Model	Parameters (M)	Precision	Recall	F1-Score	Accuracy (%)	Loss
ResNet-50	25.6	0.58	0.57	0.57	57.25	1.2706
EfficientNet-B0	5.3	0.66	0.65	0.66	66.30	1.2281
ViT-B/16	86.5	0.70	0.69	0.70	70.65	0.6053
**Proposed ResViT**	**32.1**	**0.73**	**0.74**	**0.67**	**74.09**	**0.5260**

The comparative results in Table 5 demonstrate that the proposed ResViT model consistently outperforms baseline CNN- and transformer-based architectures across all key metrics. While ResNet-50 excels at capturing local visual features, it lacks the ability to model long-range dependencies. Conversely, ViT-B/16 effectively captures global context but struggles with fine-grained visual details. EfficientNet-B0 offers computational efficiency but at the cost of reduced accuracy. In contrast, the hybrid ResViT framework successfully combines the complementary strengths of convolutional and transformer mechanisms, achieving superior generalization and enhanced sensitivity to MCI-specific patterns.

**Fig. 10:**
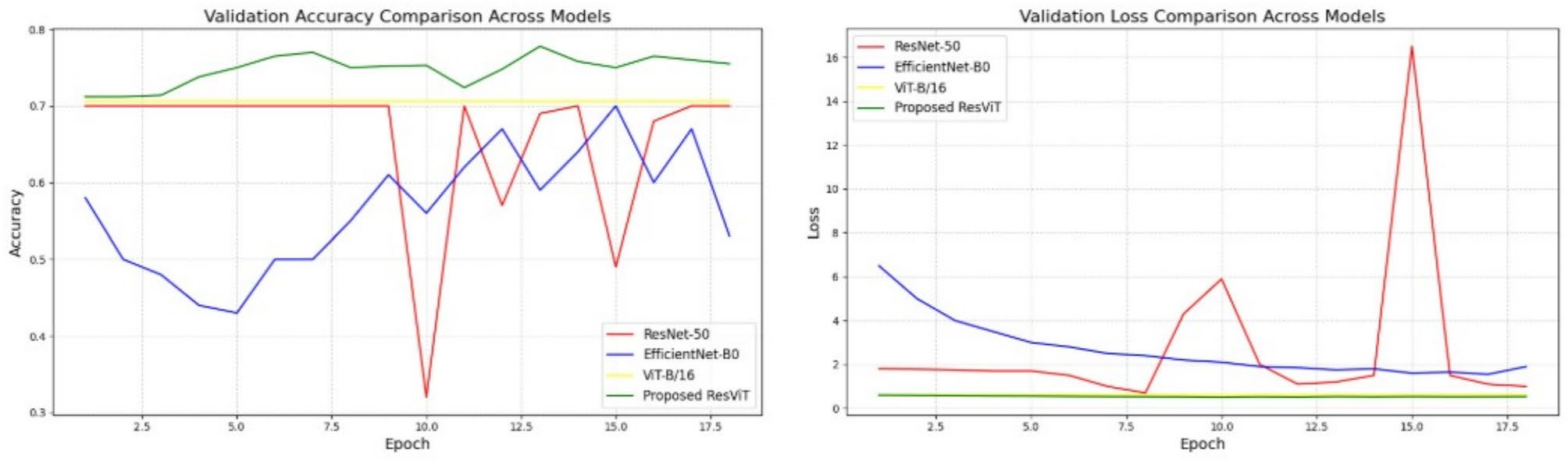
Combined Validation accuracy and loss curves for all models (ResNet-50, EfficientNet-B0, ViT-B/16, and the proposed ResViT). The ResViT model demonstrates faster convergence and higher stability compared to all baseline architectures.

To facilitate a more direct and comprehensive visual comparison across all architectures, a consolidated performance plot was generated, as illustrated in Fig. 10. This figure integrates the training and validation accuracy and loss curves for all models. It is evident that the proposed ResViT model achieves higher validation accuracy and faster convergence than the baseline architectures, reinforcing the effectiveness of its hybrid local–global feature fusion strategy. The inclusion of this comparative visualization further enhances interpretability by enabling side-by-side examination of each model’s learning dynamics within a unified graphical representation.

### Qualitative evaluation

**Fig. 11:**
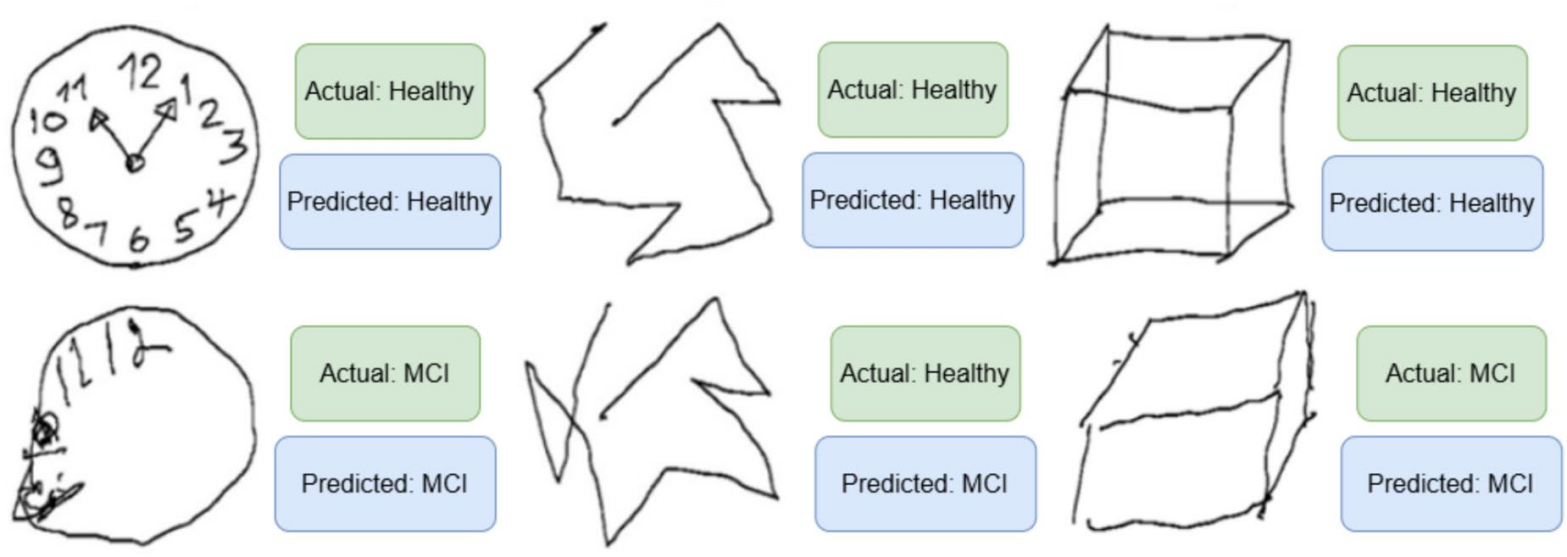
The final classification stage of the ResVit model takes the fused features from both ResNet50 and ViT-B/16 and passes them through fully connected layers to generate the output prediction. Based on the cognitive drawing inputs (Clock, Cube, Trail), the model classifies each case as either Healthy or Mild Cognitively Impaired (MCI), assisting in early detection of cognitive decline.

To better understand the decision-making process of the ResViT model, a qualitative analysis was conducted by visualizing the predictions on random test samples. Figure 11 presents several examples of clock, cube, and trail drawings, alongside their predicted and actual labels. The visualizations highlight scenarios where the model correctly classified subtle impairments and where it struggled—particularly in cases with ambiguous or minimally distorted patterns. These insights provide direction for future improvements, such as incorporating attention heatmaps or domain-specific features.

### Model interpretability and visualization

**Fig. 12:**
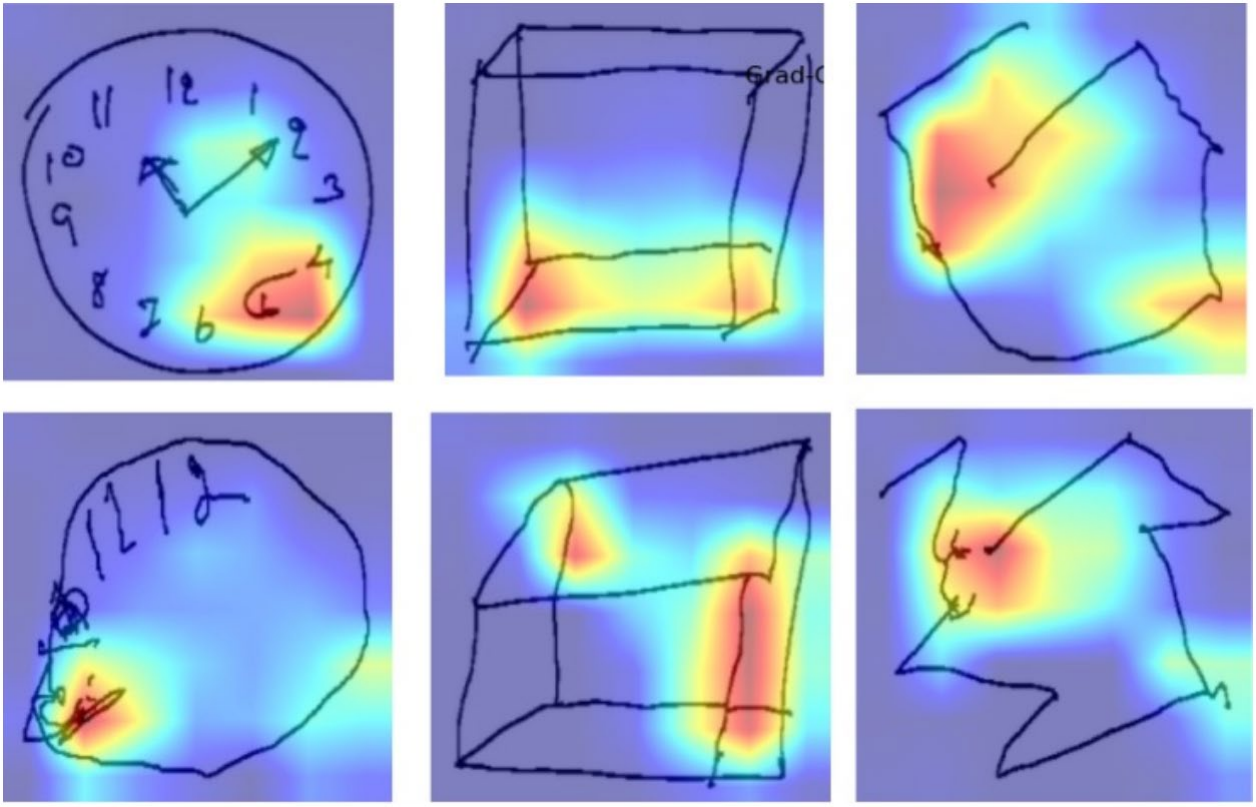
Grad-CAM visualizations of the proposed ResViT model across different cognitive drawing tasks. The attention heatmaps highlight regions that most contributed to MCI classification, illustrating the model’s focus on clinically relevant structures such as digits, edges, and trail paths.

To enhance the clinical interpretability of our framework, Grad-CAM was employed to visualize the regions that most influenced the model’s predictions. Figure 12 presents heatmaps overlaid on the Clock Drawing, Cube Copying, and Trail Making test images. The highlighted regions correspond to clinically meaningful features such as clock digits, cube corners, and trail junctions, illustrating that the ResViT model effectively captures task-specific cognitive representations. These attention patterns align closely with expert clinical assessment criteria, thereby reinforcing the model’s explainability and its potential utility in automated MCI evaluation.

## Conclusion

This study demonstrates that the integration of ResNet and Vision Transformer architectures enhances the detection of early indicators of cognitive decline. Through the analysis of multiple drawing-based cognitive tasks, the proposed ResViT model effectively captures both fine motor precision and global spatial patterns, which are commonly impaired in individuals with Mild Cognitive Impairment (MCI). While the model isn’t perfect, struggling with imbalanced data and a narrow age range it highlights the potential of AI to make cognitive screening faster and more consistent. Future work could expand datasets to include younger adults and integrate educational background data, helping the model adapt to diverse populations. Ultimately, tools like ResViT could empower clinics with low-cost, automated screenings, catching warning signs earlier and giving families more time to plan. The path forward isn’t just about better algorithms, but about building systems that understand the real-world complexity of human health.

## Data Availability

The dataset used in this study is publicly available at MCI-multiple-drawings dataset. The authors did not generate this dataset; it was obtained from https://github.com/cccnlab/MCI-multiple-drawingsCCCNLab.
